# The Daily Mile reduces depressive and anxiety symptoms in school-going Ugandan adolescents aged 16-17

**DOI:** 10.11604/pamj.2024.48.140.40024

**Published:** 2024-07-29

**Authors:** Davy Vancampfort, James Mugisha, Tine Van Damme

**Affiliations:** 1 Department of Rehabilitation Sciences, *Katholieke Universiteit* (KU) Leuven, Leuven, Belgium,; 2 University Psychiatric Centre, *Katholieke Universiteit* (KU) Leuven, Leuven-Kortenberg, Belgium,; 3 Department of Sociology and Social Administration, Kyambogo University, Kampala, Uganda

**Keywords:** Anxiety, depression, physical activity

## Abstract

**Introduction:**

school-based physical activity (PA) programs such as The Daily Mile (TDM) might be vital in the prevention and treatment of mental health problems in adolescents in low-income countries. The aim of this single-arm non-controlled pilot intervention study was to investigate TDM on symptoms of anxiety and depression in adolescents aged 16-17 years in Uganda.

**Methods:**

The Daily Mile (TDM) took place between February and April 2022. In total 177 adolescents (62.7% girls) completed the Patient Health Questionnaire-9 (PHQ-9) and Generalized Anxiety Disorder-7 Questionnaire (GAD-7) pre and post-12 weeks TDM.

**Results:**

moderate effect sizes were found for reductions in PHQ-9 (Cohen´s d=-68, 95% CI=-0.84 to -0.52, P<0.001) and GAD-7 (Cohen´s d=-0.54, 95%CI=-0.68 to -0.38, P<0.001) following TDM. In those with at least mild symptoms, large effect sizes were observed for reductions in PHQ-9 (Cohen´s d=-0.94, 95%CI=-1.14 to -0.72, P<0.001) and GAD-7 (Cohen´s d =-0.85, 95% CI=-1.07 to -0.62, P<0.001) following TDM. The prevalence of mild depression dropped from 70.1% to 50.8% and moderate depression from 28.2% to 15.3%, while the prevalence of mild anxiety dropped from 54.8% to 38.4%, and moderate anxiety from 21.5% to 10.7% (all P<0.001). Rates of severe depression and anxiety did not drop significantly.

**Conclusion:**

The Daily Mile (TDM) might potentially reduce mild and moderate symptoms of anxiety and depression in school-going adolescents in low-income countries such as Uganda. For more severe cases, additional support is needed.

## Introduction

Mental health problems are worldwide a leading cause of disability [1]. In low- and middle-income countries they represent one of the most important barriers to sustainable development [2] as they prevent young people from reaching their full potential and are associated with premature mortality from suicide and other diseases [1]. The first onset of mental health problems is often during early adolescence [3]. Consequently, this period in life represents an important window for prevention and early intervention [4]. It has been questioned before whether schools can play a role in the prevention of mental health problems in adolescents with known risk factors and promotion of positive mental health for all, defined as a state of well-being where adolescents can cope with the normal stresses of life and successfully participate in everyday life [5]. In low- and middle-income countries such as Uganda, schools could, in particular, be an effective setting to reach adolescents and their families at no extra cost since there are no clear pathways for screening and access to mental health interventions, mainly due to a lack of facilities and skilled human resources [6-8].

The need is however very high in this part of the world [9,10]. The prevalence for mild-to-moderate depressive symptoms in school-going Ugandan adolescents aged 14-17 years ranges from 21% [9] to 30% [10], while during the COVID-19 pandemic this rate even rose up to 56% [11]. Similarly, during COVID-19 the prevalence for mild-to-moderate symptoms of generalized anxiety was as high as 47%.

Risk factors for developing mental health problems are older age, i.e. 16 years and above, and being physically inactive [9-11]. A focus on physical inactivity is particularly relevant in Uganda since very recent evidence demonstrated that 86% of school-going adolescents are physically inactive [12].

Because adolescents spend a significant amount of time in school and schools provide access to large populations from a range of socioeconomic backgrounds, schools should be targeted as a key setting to improve adolescents´ overall physical activity levels. It is known from studies in high-income countries that school-related physical activity interventions improve resilience, positive mental health, and well-being and reduce anxiety [13]. Considering the beneficial effects of physical activity in high-income countries, there is sufficient reason to explore school-based initiatives to increase physical activity for mental health in low-income countries such as Uganda as well. Data for Uganda, and by extension for low-income countries, are however currently lacking. A potentially feasible and low-cost school-based physical activity intervention such as The Daily Mile (TDM) program has to the best of our knowledge not been investigated before in low-income settings, neither for mental and physical health. The aim of TDM is that participants perform at least a one-mile outdoor run or walk at a self-selected pace during school hours [14]. Teachers can freely choose the timing during the school day, but it is not supposed to replace physical education classes, break times, or after-school activities [15]. The intervention´s simple methodology and replicability have resulted in rapid uptake and TDM is now being delivered in more than 14,000 schools across more than 85 countries worldwide [16]. Previous research in high-income countries has already demonstrated the safety, acceptability, and feasibility of TDM [17]. Moreover, significant increases in physical fitness components [14,17-19] and physical activity and reductions in sedentary behaviour [14,20] have all been documented. The efficacy of TDM on cognitive functioning is, to date, rather mixed [20-23]. Despite the growing body of evidence, there is only preliminary evidence on the mental health outcomes of TDM in school-going adolescents, and recently calls were made to explore these mental health outcomes of TDM in more detail [15,23-26].

To fill the current gaps in the literature, this study aimed to examine whether TDM reduces symptoms of depression and anxiety in Ugandan school-going adolescents. First, we will investigate whether TDM reduces symptoms of depression and anxiety including all participating school-going adolescents in the analyses. Second, we will investigate whether the prevalence rates of mild, moderate, and severe depressive symptoms and of mild, moderate, and severe anxiety symptoms will reduce following TDM. Third, we will explore whether TDM reduces symptoms of depression and anxiety in those at risk, i.e. with at least mild symptoms of anxiety or depression. As school-going adolescents aged 16 years or older are at risk for developing mental health problems [9-11], we focused on this particular age group. We hypothesize that symptoms of depression and anxiety will reduce following TDM in Ugandan school-going adolescents aged 16 or 17 years, and effects will be larger in those presenting with at least mild symptoms.

## Methods

**Study design:** this is a single arm, non-controlled pilot intervention study.

**Study setting:** this study took place in the largest secondary mixed (boys and girls) private, boarding school in Mpigi District situated in Central Uganda (i.e. Sint Maria Goretti Secondary School Katende). The population in this district is predominantly semi-literate (80%), and involved in subsistence farming (59%) [27].

**Study population:** included were all students aged 16 or 17 years from the private, boarding school in Mpigi District. Student aged 18 years or older could participate as this was a real-world intervention but they were excluded from the analyses. There were no other exclusion criteria.

**Study sampling:** this school was chosen by the health district office as it was the only one fully operational during the planning stage of this study which took place during the COVID-19 pandemic.

**Study intervention:** within a 12-week TDM intervention, teachers could freely choose the timing during the school day to perform the activity. Participants were encouraged to run or walk at least one mile outside (approximately 15-20 minutes) at a self-selected pace, wearing normal school clothes and shoes. The school was instructed and encouraged to implement TDM every school day, i.e. 6 days per week. TDM was not supposed to replace physical education classes, break times, or after-school activities [15]. Physical education was however not part of the curriculum of the students. Consequently, TDM was the only physical activity offered during the intervention period. After school hours, students are however able to use the sports field and playground of the school.

### Study variables

**Patient Health Questionnaire-9 - adolescent version (PHQ-9):** the PHQ-9 is the nine-item depression scale of the Patient Health Questionnaire, which can be used in both adolescent and adult populations [28]. Each item asks the individual to rate the severity of his or her symptoms over the past two weeks. Each item is scored on a Likert scale with symptoms rated as 0 (not at all), 1 (several days), 2 (more than half the days), and 3 (nearly every day). The total score ranges from 0 to 27. Higher scores implicate more severe symptoms. The optimal cut-off for the presence of mild depressive symptoms is ≥5, for moderate depressive symptoms ≥10, and for severe depressive symptoms ≥20 [28]. The PHQ-9 has been validated for assessing depression in rural Uganda [29].

**Generalized Anxiety Disorder-7 (GAD-7):** the Generalized Anxiety Disorder Assessment (GAD-7) [30] is a seven-item instrument that is used in both adolescent and adult populations to measure or assess the severity of anxiety symptoms. Each item asks the individual to rate the severity of his or her symptoms over the past two weeks. Each item is scored on a Likert scale with symptoms rated as 0 (not at all), 1 (several days), 2 (more than half the days), and 3 (nearly every day). The total score ranges from 0 to 21. Higher scores implicate more severe symptoms. The optimal cut-off for the presence of mild anxiety symptoms is ≥5, for moderate anxiety symptoms ≥10, and for severe anxiety symptoms ≥15 [30]. The GAD-7 has been used before in Uganda [31].

**Study procedure:** The Daily Mile (TDM) took place between February and April 2022 when schools in the country were reopening after the second COVID-19 wave. All adolescents at senior 3 from the included school were invited to participate. Since English is the official/language used in secondary schools in Uganda, the interviews were executed in English and not in any of the local languages. In case adolescents were willing to participate in the study, they completed the interviewer-administered PHQ-9 and GAD-7 in the week before TDM started, i.e. the baseline assessment (T1). In case adolescents were not willing to participate, no questions were completed during the assessment phase, but adolescents took part in TDM as part of the school activities. Adolescents were informed that non-participation in the assessments would not affect their relationship with the school. After the 12 weeks of TDM, all participating adolescents completed the interviewer-administered PHQ-9 and GAD-7 in the week following the intervention, i.e. post-intervention assessment (T2).

**Statistical analyses:** continuous data were tested for normality using the Shapiro-Wilks test and PHQ-9 and GAD-7 scores were found to be normally distributed. All data are therefore presented as means and standard deviations. Paired t-tests were used to investigate differences in PHQ-9 and GAD-7 scores pre- (T1) versus post (T2)-intervention. Within-group effect sizes for changes between T1 and T2 in PHQ-9 and GAD-7 scores were calculated using Cohen´s d based on the pooled standard deviations [32]. The criteria for the magnitude of the effect sizes were as follows: small = 0.20-0.49, medium = 0.50-0.79, and large ≥0.80 [32]. Differences in pre-(T1) versus post (T2)-intervention prevalence rates of mild, moderate, and severe depressive symptoms and of mild, moderate, and severe anxiety symptoms were investigated with McNemar´s tests. Sensitivity analyses were performed exploring differences in PHQ-9 and GAD-7 scores pre-(T1) versus post-(T2)-intervention in those with at least mild symptoms, i.e. in those with PHQ-9≥5, and in those with GAD-7≥5. The significance level was set at P<0.05. Statistical analyses were performed using the statistical package SPSS version 28.0 (SPSS Inc., Chicago, IL).

**Ethical considerations:** adolescents were informed that non-participation would not affect their relationship with the school. All participants gave their written informed assent and the legal representatives of the participating adolescents their written informed consent. Participants were not financially compensated but received a bottle of water and a chapati (wheat bread) following each session and during the completing of the interviewer-administered Patient Health Questionnaire-9 - adolescent version (PHQ-9) [28] and the Generalized Anxiety Disorder-7 questionnaire (GAD-7) [30]. The study was approved by the ethical committee of Mengo Hospital (MH/REC/134/09-2022).

## Results

**Participants:** in total, 182 adolescents agreed to participate. None of the students refused to participate. Two students who participated did not obtain written informed consent from their legal representatives and were therefore excluded from the analyses. None of the participants dropped out due to medical or motivational reasons. Three students were not at school during the post-intervention test days and had to be excluded. Therefore, 177 students aged 16 or 17 years (111 girls and 66 boys) did complete the interviewer-administered questionnaires. There were no missing data. No adverse events were reported.

**Differences pre- versus post-TDM in depression, anxiety, and physical activity levels among Ugandan school-going adolescents aged 16 and 17 years:** Table 1 provides an overview of the pre-post intervention differences. Significant, moderate effect sizes were found for reductions in PHQ-9 and GAD-7 scores. At the individual level, post versus pre-test scores on the PHQ-9 ranged from -13 to +6. In 12 adolescents, we did observe an increase in depression post versus pre-intervention, in 36 no change, and in 129 adolescents a reduction. With regards to changes in anxiety symptoms, at the individual level, post versus pre-test scores on the GAD-7 ranged from 18 to +8. In 10 adolescents, we did observe an increase in anxiety symptomatology post versus pre-intervention, in 47 no change, and in 120 adolescents a reduction.

**Table 1:** differences in levels of anxiety and depressive symptoms among 177 Ugandan school-going adolescents aged 16 and 17 years pre- versus post-TDM

	Pre-TDM mean ± SD	Post-TDM mean ± SD	P	Effect sizes	95% CI
PHQ-9 total score	7.0±4.3	5.0±3.8	<0.001*	Cohen’s d = -0.68	-0.84 to -0.52
GAD-7 total score	6.0±4.2	4.4±3.6	<0.001*	Cohen’s d = -0.54	-0.69 to -0.38

* Significant when P<0.05 using paired t-tests; GAD-7: generalized anxiety disorder - 7; PHQ-9: patient health questionnaire - 9; SD: standard deviation; TDM: The Daily Mile

**Differences in prevalence of mild, moderate, and severe anxiety and depression in Ugandan school-going adolescents aged 16 and 17 years pre- versus post-TDM:** the prevalence of scoring above the threshold for at least mild symptoms of depression (PHQ-9≥5) dropped significantly following TDM from 70.1% (n=124) to 50.8% (n=90) (P<0.001). For at least moderate levels of depression symptoms (PHQ-9≥10), the prevalence dropped significantly from 28.2% (n=50) to 15.3% (n=27) (P<0.001), and for severe levels of depression (PHQ-9≥20) the prevalence dropped from 0.6% (n=1) to 0% (n=0) (P>0.99). Similarly, the prevalence of scoring above the threshold for mild symptoms of anxiety (GAD-7≥5) dropped significantly following TDM from 54.8% (n=97) to 38.4% (n=68) (P<0.001). For moderate levels of anxiety symptoms (GAD-7≥10), the prevalence dropped significantly from 21.5% (n=38) to 10.7% (n=19) (P<0.001), and for severe levels of anxiety (GAD-7≥15) from 2.8% (n=5) to 0.6% (n=1) (P=0.12).

**Differences pre- versus post-TDM in depression and anxiety levels among Ugandan school-going adolescents aged 16 and 17 years with at least mild symptoms:** in those with at least mild depression (PHQ-9≥5, n=124), a large reduction in PHQ-9 score (Cohen´s d = -0.94, 95% CI = -1.14 to -0.72, P<0.001) was found following TDM. Similarly, in those with at least mild anxiety (GAD-7≥5, n=97), a large reduction in GAD-7 score (Cohen´s d = -0.85, 95% CI = -1.07 to -0.62, P<0.001) was found following TDM.

## Discussion

The current pilot study is the first to explore the efficacy of TDM in school-going adolescents in a low-income country. Our data show that following 12 weeks of TDM, at school level, lower levels of anxiety and depression can be observed among school-going adolescents in a rural area in Uganda, while the prevalence of mild and moderate depression and anxiety significantly dropped, but not for severe depression and anxiety. Of interest from a practical perspective is that TDM seems to be a feasible school-based physical activity intervention for low-income settings. No drop-outs and no adverse events were reported. However, the latter finding should be interpreted with caution since participants received a bottle of water and a chapati after each TDM session. The current findings are important, in particular considering the high burden of depression and anxiety among school-going adolescents in Uganda. Previous studies did demonstrate that the COVID-19 pandemic had a serious mental impact on young people in Uganda as well, even more so in those who were not attending schools [33,34].

Our data furthermore demonstrate that during TDM only in very few adolescents, levels of anxiety or depression increased providing preliminary evidence for potential preventive effects of TDM, while effect sizes were largest in those with at least mild symptoms providing, in its turn, preliminary evidence for potential treatment-related effects. Although to the best of our knowledge, worldwide no data are available exploring the anxiolytic and antidepressant effects of TDM in school-going adolescents, the findings are in line with the emerging global mental health-informed physical activity literature. It has been demonstrated before that physical activity has preventive and curative effects on anxiety and depression in adolescents [35-40]. However, our data indicate as well that TDM is not able to reduce severe depression and anxiety and for this risk group, additional efforts are needed.

The current findings should however be confirmed in controlled trials. Future research is also needed to explore potential underlying mechanisms for any mental health effects of TDM on school-going adolescents in low-income countries. Previous studies focusing on the benefits of physical activity investigated mainly moderate-to-vigorous intensity physical activity in adolescents from upper-middle- and high-income countries. The current evidence indicates there are several neurobiological and psychosocial pathways that could clarify the observed reduction in mental health symptoms when being more physically active. For example, neurobiological changes such as an increased cerebral blood volume and/or flow and changes in peripheral biomarkers such as an increase in circulating growth factors, and anti-inflammatory markers have been reported before [41]. From a psychosocial perspective, physical activity provides adolescents an opportunity for social interaction (relatedness), mastery in the physical domain (self-efficacy and perceived competence), and improvements in appearance self-perceptions (self-esteem and body image), and independence (autonomy) [42]. In particular, the psychosocial mechanisms might be relevant to explore in the context of TDM. A recent study from Wales, UK [43] did show that adolescents who participate in TDM do often report social benefits such as the opportunity to interact with peers and the feeling of belonging to a group or a team during the activity. TDM can be regarded as an activity in which every participant can perform at one´s own pace in a group. With regards to self-efficacy, perceived competence, and autonomy, existing theoretical models such as the exercise and self-esteem model [44] demonstrate that participating at one´s own pace may improve self-efficacy, which generalizes to one´s physical self-concept and consequently, further to global self-esteem. Finally, also the fact that TDM can be performed outdoors might improve mental health. A previous review demonstrated that compared with exercising indoors, being physically active in natural environments is associated with greater feelings of revitalization and positive engagement, decreases in tension, confusion, anger, and depression, and increased energy, while participants report greater enjoyment and satisfaction with outdoor activities [45].

**Implications for school mental health policies:** the current data indicate that there is evidence for school health policy makers to consider implementing TDM within a whole-school health policy targeting the mental health of pupils. TDM is in particular interesting in low-resourced settings due to the simplicity of adoption and delivery by teachers, with no equipment or special training required. Our data furthermore demonstrate that TDM is also safe to implement since no adverse events were reported. Importantly, for severe cases, additional support is needed.

**Limitations and future research:** the current findings need to be interpreted in light of several methodological limitations. First, the lack of a control group limits the validity of the current study, i.e. we cannot be certain that any changes observed were actually due to TDM. Second, we only explored the outcomes of depression and anxiety immediately post-intervention. Considering these limitations, randomized controlled studies using long-term follow-up are required to confirm or refute whether TDM improves mental health outcomes in school-going adolescents in Uganda. Third, we only assessed adolescents in one private school that was operational during the COVID-19 pandemic. Therefore, the outcomes of TDM might not be representative for all adolescents in private schools, and for those in public schools. Fourth, some other potentially confounding factors were not considered in the analyses, such as the socio-economic status of the adolescents and daily levels of physical activity and sedentary behaviour. Finally, we lack data on the adherence of the participating adolescents to TDM.

## Conclusion

Our data provide preliminary evidence that school-based physical activity interventions such as TDM have the potential to prevent mild to moderate mental health problems and reduce the burden of existing mental health problems in adolescents. Interestingly, no dropouts or adverse events were reported. For more severe cases, additional support is however needed. Randomized controlled trials with long-term follow-up assessments are needed to confirm or refute the current outcomes.

### What is known about this topic

- *In low- and middle-income countries such as Uganda, schools could be an effective setting to reach adolescents and their families at no extra cost since there are no clear pathways for screening and access to mental health interventions, mainly due to a lack of facilities and skilled human resources;*
- *The prevalence of mild-to-moderate depressive symptoms in school-going Ugandan adolescents aged 14-17 years ranges from 21% to 30%, while during the COVID-19 pandemic, this rate even rose up to 56%.*

### What this study adds

- *The Daily Mile lowers levels of mild to moderate anxiety and depression among school-going adolescents in a rural area in Uganda, while for more severe cases, additional support is needed;*
- *The Daily Mile is interesting to implement in low-resourced settings due to the simplicity of adoption and delivery by teachers, with no equipment or special training required;*
- *The Daily Mile is safe to implement.*
